# Effect of shoe heel height on vastus medialis and vastus lateralis electromyographic activity during sit to stand

**DOI:** 10.1186/1749-799X-3-2

**Published:** 2008-01-10

**Authors:** Lindsay Edwards, John Dixon, Jillian R Kent, David Hodgson, Vicki J Whittaker

**Affiliations:** 1 Walsall Teaching Primary Care Trust, Jubilee House, Bloxwich Lane, Walsall, UK; 2 School of Health and Social Care, University of Teesside, Middlesbrough, UK

## Abstract

**Background:**

It has been proposed that high-heeled shoes may contribute to the development and progression of knee pain. However, surprisingly little research has been carried out on how shoe heel height affects muscle activity around the knee joint. The purpose of this study was to investigate the effect of differing heel height on the electromyographic (EMG) activity in vastus medialis (VM) and vastus lateralis (VL) during a sit to stand activity. This was an exploratory study to inform future research.

**Methods:**

A repeated measures design was used. Twenty five healthy females carried out a standardised sit to stand activity under 4 conditions; barefoot, and with heel wedges of 1, 3, and 5 cm in height. EMG activity was recorded from VM and VL during the activity. Data were analysed using 1 × 4 repeated measures ANOVA.

**Results:**

Average rectified EMG activity differed with heel height in both VM (F_2.2, 51.7 _= 5.24, p < 0.01), and VL (F_3, 72 _= 5.32, p < 0.01). However the VM: VL EMG ratio was not significantly different between conditions (F_3, 72 _= 0.61, p = 0.609).

**Conclusion:**

We found that as heel height increased, there was an increase in EMG activity in both VM and VL, but no change in the relative EMG intensity of VM and VL as measured by the VM: VL ratio. This showed that no VM: VL imbalance was elicited. This study provides information that will inform future research on how heel height affects muscle activity around the knee joint.

## Introduction

Patellofemoral pain syndrome (PFPS) and osteoarthritis (OA) of the knee are common musculoskeletal conditions [1-6]. Both PFPS and OA knee are more prevalent in females than males [1,2]. Although the pathomechanics of these pathologies may differ, it is believed that muscle dysfunction is a contributing factor in both. In particular, the proposed imbalance between the quadriceps muscles vastus medialis (VM) and vastus lateralis (VL) is believed to be important, and this has been investigated in patients with PFPS [7-13] and also OA knee [14,15] using electromyography (EMG). Either a delay in EMG onset timing or a reduced EMG intensity in VM relative to VL may lead to a biomechanical imbalance at the patellofemoral joint [16], and patellofemoral malalignment has been suggested to be one of the major causes of PFPS [3,4,6,17].

It has been proposed that high-heeled shoes may contribute to the development and progression of knee OA [18,19]. In a recent survey [20], the American Podiatric Medical Association ascertained that 62% of American women wear heels over two inches in height regularly and that these are considered high heels. As this is a possible risk factor that may contribute to knee pathologies in women, and one that can be modified, it warrants attention. However, this area has received surprisingly little consideration. Despite the higher prevalence of PFPS and OA knee in females compared to males, how shoe heel height affects VM and VL EMG activation, and their relative levels as measured by the VM: VL ratio, has not been investigated.

There is some evidence to suggest that shoe heel height may affect muscle activation. Hertel et al [21] reported that lateral and medial orthotics increased EMG activity in VM and decreased EMG activity in VL. High heels have been shown to elicit greater activity in rectus femoris, and cause larger vertical and anterior-posterior ground reaction forces during gait [22], and also to increase erector spinae and tibialis anterior EMG activity [23]. In addition, it has been reported that high-heeled shoes increase the external adduction moment at the knee joint [19], implying an increased medial compartment load. This may affect muscle activity around the knee joint, and theoretically could manifest as either an increase in VM activity, as observed by Hertel et al [21] above, or a reduction in VM activity from inhibitory mechanisms elicited by altered biomechanical forces at the knee joint.

Much of the above research focuses on gait, because it is appreciated that repetitive loading is an important factor in these knee pathologies. However it has been observed that VL activity is not affected by wearing heels during gait [23]. In contrast, the effect of heel height on VM and VL muscle activation during sit-to-stand has not been studied. As this is a task with greater muscle demand than gait, it is possible that any effect of heel height on muscle activation patterns may be greater and more detectable than in gait.

The aim of this exploratory study was therefore to investigate the effect of differing heel height on the EMG activity in VM and VL during sit to stand. It was hypothesised, because of possible alterations to mechanical alignment, stability and moments at the knee joint, and somatosensory afferent signalling, that increasing heel height would elicit increased VM activity, relative to that of VL, to stabilise the patellofemoral joint.

## Methods

An exploratory repeated measures study was carried out.

### Participants

Twenty five healthy females participated in the study, mean (SD) age 24.4 (2.1) years, height 1.65 (0.07) m, body mass 64.2 (11.5) kg, BMI 23.5 (3.7) kg/m^2^. Thirty-one females were recruited for this study but six were excluded due to recent knee pathologies. These were selected, using convenience sampling, from the female population of the University of Teesside, accessed through email, targeting the MSc and BSc physiotherapy courses. Participants had to be female, and accustomed to wearing high heels, although not necessarily on a daily basis, in agreement with previous literature [22]. Participants were excluded if they had chronic ankle or knee problems, or had experienced ankle or knee injuries in the previous twelve months. The School of Health and Social Care Ethics Committee of the University of Teesside granted ethical approval for the study. All participants gave informed written consent to participate in the study.

### Instrumentation and procedure

Surface EMG (BIOPAC Inc., USA) was used to measure the activity of VM and VL. The BIOPAC system comprised an MP100 data acquisition unit, and high level transducer HLT100 coupled to a universal interface module UIM100C. EMG signals were digitised, stored and analysed using AcqKnowledge software version 3.5.3.

After cleaning the skin with isopropyl alcohol, active surface EMG recording electrodes (BIOPAC Inc., USA, TSD150B, Ag/Ag Cl, diameter 11.4 mm, electrode spacing 20 mm centre to centre, with a built in 350× amplification and a 3 dB bandpass of 12 to 500 Hz) were placed on VM and VL at standardized sites on the dominant leg, determined as the one with which they would kick a ball. The electrodes were oriented in the estimated direction of the muscle fibres [24]. The VM electrode was positioned 4 cm superior to and 3 cm medial to the superomedial border of the patella, and orientated 55° to the femur [13]. The electrode for VL was situated 10 cm superior and 7 cm lateral to the superior patella border, and then oriented 15° to the femur [13]. Hypoallergenic conductive gel (Lectron II, Pharmaceutical Innovations Inc., USA) was applied to the electrodes to facilitate electrical contact with the skin surface. A ground electrode (Blue Sensor^®^) was attached to the contralateral tibial tuberosity. All electrodes were taped to the skin to reduce movement artifacts and remained in place throughout the study. The EMG data were recorded at a sampling rate of 1000 Hz.

**Figure 1:**
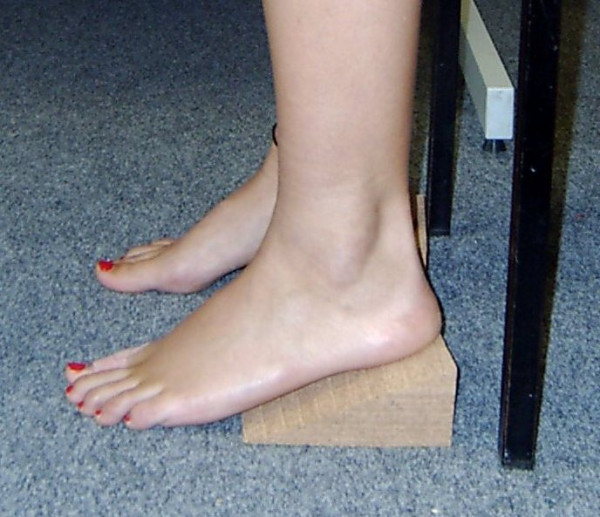
Cork wedge methodology used.

Participants carried out three repetitions of a sit to stand task under each of four conditions; barefoot, and with heel heights of 1 cm, 3 cm and 5 cm. In order to mimic shoes with these different heel heights, cork wedges of these specific heights were constructed. Cork wedge or heel block methods have been used in previous studies [25,26]. Despite having some limitations [26], this method allowed us to overcome methodological issues associated with the standardisation of heel height when participants wear their own shoes [19]. To establish an approximate size, four females with shoe sizes varying from size 4 to 8 had their feet measured. The wedges were made 12 cm long to approximate average foot size of the participant group. They had a wide base as these are considered the most sensible amongst women of any age [19]. A mean width of 10 cm was established, and to allow for variation the wedges were constructed 12 cm wide. Three heights were constructed 1 cm, 3 cm and 5 cm, with the 5 cm constituting the high heel height in agreement with Gefen et al. [27]. The lowest height of 1 cm has been described as equivalent to a typical shoe heel elevation [22]. A middle height was chosen to establish any changes between the heights. An example of the wedge is shown in Figure 1.

Each participant was requested to remove shoes and socks to maintain safety. The order in which the heel height conditions were tested was randomised by allowing the participants to choose a numbered card prior to each condition. The cards were numbered 1 to 4, relating to the 4 conditions, placed face down, and the choice of card order indicated the condition order. Participants were seated on a standard chair of height 47 cm, with their feet a comfortable width apart. Foot position was kept the same between tests. For the wedge conditions, every participant was positioned on the cork wedges so that they mimicked wearing heels, with the forefoot and toes on the floor and the heel at the back of the wedge. They were then asked to stand, in their own time without using their arms to assist, and remain standing until requested to sit. The subjects remained standing for approximately 30 seconds before being asked to sit. After five seconds of sitting they were asked to stand again, using the same instructions. Participants carried out three sit to stands for each condition.

### Data processing

In this study, EMG normalisation was not required because the participants acted as their own control and all procedures were performed in the same session, without the electrode positions being altered [28]. Using the AcqKnowledge 3.5.3 software, a 20 Hz high pass filter was firstly applied to the raw data to remove any movement artifacts and then the raw EMG was processed using a root mean square moving window of 50 ms duration [29,30]. For each participant, the average rectified value (ARV) [31] was calculated for VM and VL in each sit to stand repetition by dividing the EMG integral by the contraction time interval. To determine the cut-off points for each EMG burst, the onset was determined as the point at which the signal exceeded the mean resting value of a 300 ms window prior to activity, by more than 3 standard deviations for over 30 ms [32,33], and the cessation point was the point at which the signal was less than or equal to the mean resting value plus 3 standard deviations of a 300 ms window after standing for more than 30 ms. It was necessary to use two separate thresholds, as often once participants were standing, having finished the sit to stand, the EMG signal did not quite return to the threshold for onset, the quadriceps being very slightly active in standing, as has been previously reported [34]. The data were visually checked to ensure artifacts were not incorrectly identified as onsets. The EMG ARV values of VM and VL were then averaged over the three repetitions for each condition. In addition, the mean VM and VL EMG ARV data for each participant were then used to calculate the ratio of VM: VL EMG activity for each condition.

### Statistical analysis

Data were analysed using the Statistical Package for Social Sciences (SPSS) version 11.5. The separate EMG ARV data for VM and VL, and the data for the VM: VL ratio were all tested for statistical significance. For each of these variables, a 1 × 4 repeated measures analysis of variance (ANOVA) was carried out to determine statistically significant differences between the four conditions. The level of statistical significance was set at 0.05. Where the assumption of sphericity was violated, a Greenhouse-Geisser correction was applied. Where the ANOVA showed a significant difference, post hoc pairwise comparisons were used to identify where specific differences occurred, with Bonferroni adjustments for the use of multiple comparisons. Additionally, intraclass correlation coefficients (ICC 3, 3) were calculated for the ARV of VM and VL to assess the repeatability of the 3 repetitions during each of the four conditions.

## Results

**Figure 2:**
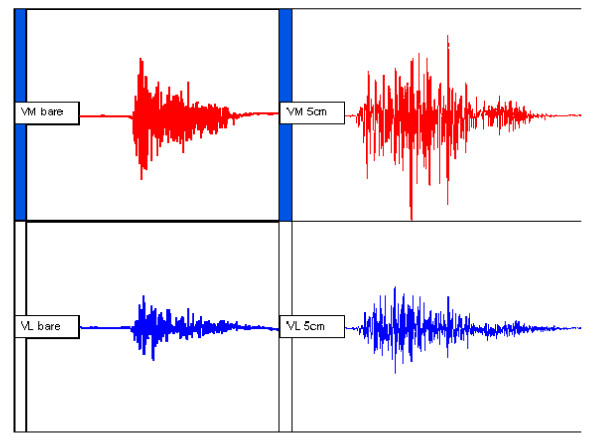
Representative raw electromyographic data during sit to stand. Left trace shows barefoot condition, right trace shows 5 cm heel height.

A typical EMG trace showing raw VM and VL muscle activity during sit to stand in the barefoot and 5 cm condition is displayed in Figure 2. The mean (SD) EMG ARV data for VM and VL are shown in Table 1. The EMG activity was significantly different by condition for VM (F_2.2, 51.7 _= 5.24, p < 0.01), and for VL (F_3, 72 _= 5.32, p < 0.01). The mean differences and 95% confidence intervals between the conditions are presented in Table 2. The pairwise comparisons with Bonferroni adjustments revealed that for VM the difference between barefoot and 5 cm heels was statistically significant (p < 0.01). For VL there were statistically significant differences between barefoot and 3 cm heels (p < 0.01) and barefoot and 5 cm heels (p < 0.05).

**Table 1:** Average rectified values of EMG activity of VM and VL during sit to stand

	Mean (Standard Deviation) EMG activity (μV)	
Heel Height	VM	VL

Barefoot	84.5 (53.7)	52.5 (28.0)
1 cm	96.4 (55.4)	57.3 (26.5)
3 cm	104.2 (68.2)	61.3 (32.5)
5 cm	102.5 (60.5)	62.0 (29.9)

**Table 2:** Mean (95% confidence interval) difference between conditions in average rectified values of EMG activity (μV) of VM and VL during sit to stand

Comparison	VM	VL
Barefoot v 1 cm	11.9 (-3.6 to 27.4)	4.9 (-3.2 to 13.0)
Barefoot v 3 cm	19.8 (-1.6 to 41.1)	8.8 (2.1 to 15.6) **
Barefoot v 5 cm	18.0 (5.8 to 30.3) **	9.5 (1.3 to 17.8) *
1 cm v 3 cm	7.8 (-9.2 to 24.8)	3.9 (-3.7 to 11.6)
1 cm v 5 cm	6.1 (-7.5 to 19.7)	4.6 (-3.6 to 13.0)
3 cm v 5 cm	-1.7 (-15.6 to 12.1)	0.7 (-6.5 to 8.0)

*Significant at p < 0.05, **Significant at p < 0.01

**Figure 3:**
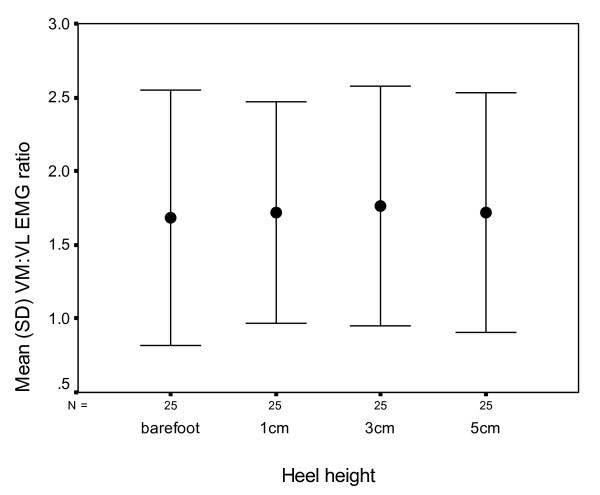
Mean vastus medialis: vastus lateralis average rectified EMG ratios during sit to stand under the four conditions.

The mean (SD) VM: VL EMG ARV ratios were 1.68 (0.87) for the barefoot condition, 1.72 (0.75) for 1 cm heels, 1.76 (0.81) for 3 cm heels, and 1.72 (0.81) for 5 cm heels, as shown in Figure 3. The repeated measures ANOVA revealed that the difference between the conditions in the VM:VL ratio was not statistically significant (F_3, 72 _= 0.61, p = 0.609).

The ICC analysis revealed high repeatability for the three ARV values of VM and VL during each condition, with all ICC (3, 3) values being 0.9 or greater. The ICC (3, 3) values ranged from 0.90 for VM in the 1 cm heel height to 0.96 for VM in the 3 cm heel height.

## Discussion

The results of this study showed that increasing heel height caused increases in EMG activity in both VM and VL that were statistically significant in certain conditions. The 1 cm heel did not elicit significantly greater EMG intensity than the barefoot condition in either VM or VL. For VL the increase at 3 cm and 5 cm reached statistical significance, as did the increase at 5 cm for VM. However, heel height did not significantly affect the VM: VL EMG ratio, indicating that the relative activity in both muscles was similar. These results therefore show that carrying out a sit to stand task wearing high heels requires greater muscle activation in both VM and VL, but there is no evidence that this causes any significant imbalance between VM and VL.

A comparison of the results of the present study with those previously published is interesting. To the authors' knowledge, no studies of heeled gait have evaluated both VM and VL activity, and only two have investigated any quadriceps muscle EMG activity [22,23]. Stefanyshyn et al. [22] evaluated EMG in rectus femoris during gait, and reported that this was significantly increased with heel height increases. Lee et al. [23] measured EMG of VL and observed that the effect of increasing heel height on EMG during gait was not statistically significant. Differences in the relative levels of EMG activity in VM and VL have been found to result from alterations in normal knee mechanics. Hertel et al. [21] evaluated the effect of orthotics rather than normal shoe heel height. During single leg squat and lateral step down activities, it was found that orthotics increased EMG intensity in VM and gluteus medius, but not in VL. Physiotherapeutic patellar taping has been shown to increase the VM:VL ratio during a squat [35]. Studies of experimental knee effusion have observed greater levels of inhibition in VM than VL [36-38]. Had the present study found an alteration or imbalance in the VM: VL ratio when wearing heels, this could have been a mechanism by which heels were an influencing factor in knee pathologies.

The results of the present study provide some clinically relevant information on how muscle activation strategies are affected by heel height. A VM: VL imbalance is understood to be a major factor in PFPS [5], and it is worthy of note that no imbalance in the VM: VL ratio was elicited by change of heel height. In addition, an increased internal knee abduction moment may play a role in development of PFPS [17]. Larger internal moments, generated by muscle or soft tissue forces on the lateral aspect of the knee, may increase the lateral force on the patella and elicit pain. In contrast, in OA knee, an increased external knee adduction moment, generated by the ground reaction force and the lever arm, is associated with a greater medial compartment load that leads to medial compartment OA [39]. This external adduction moment is counterbalanced by the internal knee abduction moment. The effect of high heels on the moments at the knee is believed to be clinically important [19]. Kerrigan et al [19] suggested caution when wearing high heeled shoes but called for further research around the effect of variable heel height, using standardised controls, as we have done here. Our VM: VL results show no evidence that any medial: lateral muscle imbalance is generated with changing heel height, and therefore are not suggestive of muscle activation patterns that increase the internal knee abduction moment.

There are limitations to the current study, and areas that can be developed further. The lack of kinematic and kinetic data means that confounding variables may be present. However this is common to many EMG studies [21,33,35,40], and does not prevent them contributing to the advancement of knowledge and informing future work. The use of cork wedges to simulate shoe heel height is not a perfect model, as discussed by Franklin et al. [26], and hence this limits the generalisability of these results. Actual high-heeled shoes generally have a narrower base that affects centre of pressure and ground reaction forces [26], and would elicit far greater balance challenges for muscle coordination. Therefore it should be clear that these results relate to the effect of shoe heel height, rather than to shoe heel type. Only a single activity, sit to stand, was studied, and this should be followed up with evaluation during activities such as gait and stair descent, and also after muscle fatigue, which could influence the outcome. The group of participants observed here consisted of young asymptomatic participants and these findings may not be generalisable to older populations. Finally, this study also used a relatively small sample size, and hence the results should be treated with care, and followed up in a larger study. Of note, the VL EMG intensity was statistically significantly different from barefoot with a 3 cm heel, whereas for VM the difference from baseline did not reach significance until 5 cm. However, as the confidence interval for the VM difference at 3 cm only just crossed the null value (Table 2), this could well be due to a lack of study power, rather than a true difference in effect between the muscles. It is possible that the differences in the VM: VL ratio could reach statistical significance in a much larger study, or in sub-groups with particular characteristics. However, the altered heel height here was sufficient to elicit significantly increased activity in both VM and VL. Therefore despite these issues, this study provides information that will inform further research and add to the evidence base of how heel height affects muscle activity around the knee joint.

## Conclusion

This study found that in healthy females, as heel height increased, there was an increase in EMG activity in both VM and VL during the sit to stand activity. This was statistically significant at 3 and 5 cm for VL, but only at 5 cm for VM. No statistically significant change was observed in the relative levels of muscle activity as measured by the VM: VL ratio. Considering the proposed importance of these muscles in knee stability, and OA and PFPS, it is necessary to investigate the effect of heel height on activation of these muscles. This is especially important considering the number of females that report wearing heels over two inches in height regularly [20].

## Competing interests

The author(s) declare that they have no competing interests.

## Authors' contributions

All authors participated in the conception and design of the study. LE collected the data. LE, JD, DH and VJW carried out data analysis. All authors participated in the drafting, progress and revision of the manuscript.
